# Regulatory function of HSA-miR-186-5p on interleukin-2 expression in lumbar degenerative disc disease: a case-control study and subgroups analysis

**DOI:** 10.1007/s10143-025-04065-0

**Published:** 2026-01-15

**Authors:** C. Kaan Yaltırık, Gonca Gül Öndüç, Müge Kopuz Álvarez Noval, Mustafa Umut Etli, Caner Sarıkaya, Selvi Duman Bakirezer, Seda Güleç Yilmaz, Luay Şerifoğlu, Selçuk Özdoğan

**Affiliations:** 1 Department of Neurosurgery, Maltepe University Hospital, İstanbul, Turkey; 2 Department of Neurosurgery, Umraniye Training and Research Hospital, Istanbul, Turkey; 3 Department of Biochemistry, Faculty of Medicine, Yeditepe University, İstanbul, Turkey; 4 Department of Basic Medical Sciences, Faculty of Medicine, Yeditepe University İstanbul, İstanbul, Turkey; 5 Department Of Medical Biology, Faculty of Medicine, Yeditepe University, Istanbul, Turkey; 6 Department of Neurosurgery, Medipol University Hospital, Istanbul, Turkey

**Keywords:** Lumbar degenerative disc disease, HSA-miR-186-5p, Interleukin-2, Inflammation, Oswestry disability index, MicroRNA, Case-control study

## Abstract

Lumbar degenerative disc disease (LDDD) is characterized by persistent inflammation and extracellular matrix degradation. Emerging as main controllers in its pathogenesis are microRNAs (miRNAs) and proinflammatory cytokines. Through the JAK/STAT signalling pathway, HSA-miR-186-5p has been linked to modulating cytokine expression, including interleukin-2 (IL-2). The aim is to find the expression levels of IL-2 and HSA-miR-186-5p in LDDDpatients and investigate their possible correlation with disease degree. 110 LDDD patients and 17 healthy controls wereincluded. ELISA measured serum IL-2 concentrations, and RT-qPCRestimated miR-186-5p levels. The Oswestry Disability Index (ODI) guidedpatients into five subgroups (G1–G5). One-way ANOVA and correlationanalysis were applied in statistical comparisons. LDDD patients had notably higher IL-2 levels than controls (*p* <0.001). According to the subgroup analysis, the IL-2 concentration peaked inG2 and G3 and gradually dropped toward G5. By contrast, miR-186-5pexpression was noticeably lowered in the LDDD group (*p* < 0.001), with G1and G5 having the lowest levels found. Across groups, an inverse trend in IL-2 and miR-186-5p expression was noted. Results imply a dysregulated interaction between HSA-miR-186-5p and IL-2 in LDDD. Particularly in early and moderate stages ofdisease, miR-186-5p downregulation could help to explain increased IL-2-mediated inflammation. These biomarkers could provide information ondisease activity and present targets for new treatments.

## Introduction

A significant cause of chronic low back pain, a disorder with a significant clinical and socioeconomic impact globally, lumbar degenerative disc disease (LDDD) is progressive loss of disc matrix integrity, cellular death, and more inflammatory signalling inside the disc microenvironment are linked to the degeneration of intervertebral discs [1]. Among inflammatory mediators, interleukin-2 (IL-2), a cytokine usually associated with T-cell activation, has become a possible actor in chronic degenerative diseases since it can control immune responses in non-lymphoid tissues. Concurrently, microRNAs (miRNAs) have attracted interest for their regulating roles in extracellular matrix remodelling, inflammation, death, and other pathogenic processes. The pathogenesis of degenerative spinal diseases has been connected to changes in particular miRNAs. HSA-miR-186-5p has been demonstrated to target the IL-2 gene via the JAK/STAT pathway in neuroinflammatory environments, implying a possible function in reducing IL-2-mediated inflammation. Clinical severity corresponds with dynamic biomarker fluctuations, and previous studies have shown the relevance of miRNAs, including miR-17, in modulating cytokines like TNF-α and IL-6 in LDDD [2, 3]. However, the particular interaction between HSA-miR-186-5p and IL-2 in lumbar disc degeneration has not been studied.

Given the observed inverse relationship between miR-186-5p expression and IL-2 levels across LDDD severity subgroups and the unique biphasic expression pattern of miR-186-5p identified in this work, it is reasonable that miR-186-5p acts as a negative regulator of IL–2–driven inflammation. These molecular patterns show the most clarity in the early to moderate clinical stages, implying a possible therapeutic window.

This study thus intends to investigate the serum expression profiles of HSA-miR-186-5p and IL-2 in patients with LDDD and explore their association with clinical severity as assessed by the Oswestry Disability Index (ODI).

## Materials and methods

### Study design

The expression patterns of HSA-miR-186-5p and IL-2 in persons with LDDD and their possible correlation with clinical severity were investigated using a case-control study. Based on physical examination results, patient history, and magnetic resonance imaging (MRI), 110 individuals who had been clinically diagnosed with LDDD comprised the study cohort. The control group comprised another 17 healthy volunteers matched for age and sex. All participants were gathered from a single tertiary spine care centre between January 2024 and October 2024. The institutional review board granted ethical clearance; each participant signed written informed permission per the Declaration of Helsinki.

## Transcript extraction and sample collecting

Following an overnight fast, each subject’s antecubital vein was accessed for five millilitres of peripheral blood samples. Samples left to clot were centrifuged at 3,000 rpm for ten minutes at 4 °C. After collecting, the supernatant serum was kept at − 80 °C until needed. Following manufacturer directions, total RNA—including small RNAs—was extracted from serum using the miRNeasy Serum/Plasma Kit (Qiagen, Hilden, Germany). A NanoDrop spectrophotometer (Thermo Fisher Scientific, USA) evaluated RNA concentration and purity.

## Real-time PCR quantitative for miR-186-5p

Applied Biosystems, USA’s TaqMan MicroRNA Assay specific for HSA-miR-186-5p was used in a quantitative real-time polymerase chain reaction (qRT-PCR). Every sample underwent triplicate runs. With miR-16 acting as the endogenous reference gene, relative expression levels were computed using the 2^–ΔΔ Ct approach. Included to evaluate contamination and primer-dimer generation were no-template and no-reverse-transcription controls.

The human IL-2 enzyme-linked immunosorbent assay (ELISA) kit (R&D Systems, Minneapolis, MN, USA) allowed the measurement of IL-2 levels in serum. Means absorbance values were recorded for final analysis after all measurements were duplicated under a microplate reader set at 450 nm.

The ELISA kit had a minimum detection limit of X pg/mL and an intra-assay coefficient of variation (CV) < 10%.

To ensure data reliability, all measurements were performed in duplicate.

Hemolysis control during serum miRNA extraction was evaluated using the miR-451/miR-23a ratio to confirm sample integrity.

Patients with LDDD were stratified into five subgroups based on their Oswestry Disability Index (ODI) scores: Grade 1 (0–20%), Grade 2 (21–40%), Grade 3 (41–60%), Grade 4 (61–80%), Grade 5 (81–100%). This stratification allowed a detailed assessment of biomarker levels at several phases of disability.

### Statistical analysis

IBM SPSS Statistics version 27.0 (IBM Corp., Armonk, NY, USA) was used in data analysis. For normally distributed variables, descriptive statistics were reported as means ± standard deviations; for non-normally distributed variables, medians (interquartile range). The Shapiro-Wilk test evaluated normality. Either the Kruskal–Wallis test for non-parametric data or one-way ANOVA for parametric data tested group differences. Bonferroni correction helped to adjust post hoc pairwise comparisons. The correlation between miR-186-5p expression and IL-2 levels was ascertained using Spearman’s correlation coefficient. From all the studies, a p-value less than 0.05 was regarded as statistically significant.

## Results

### Clinical and demographic characteristics

The study included 110 patients diagnosed with lumbar degenerative disc disease (LDDD) and 17 healthy controls. The LDDD group comprised 57 men and 53 women with a mean age of 50.2 ± 8.7 years, while the control group consisted of nine men and eight women with a mean age of 48.6 ± 7.9 years. There were no significant differences between the groups regarding age or sex (*p* > 0.05). The mean Oswestry Disability Index (ODI) score for the LDDD cohort was 46.3 ± 17.5. Based on ODI scores, patients were classified into five subgroups: Grade 1 (*n* = 19), Grade 2 (*n* = 26), Grade 3 (*n* = 23), Grade 4 (*n* = 22), and Grade 5 (*n* = 20).

### Serum IL-2 levels in LDDD

**Fig. 1:**
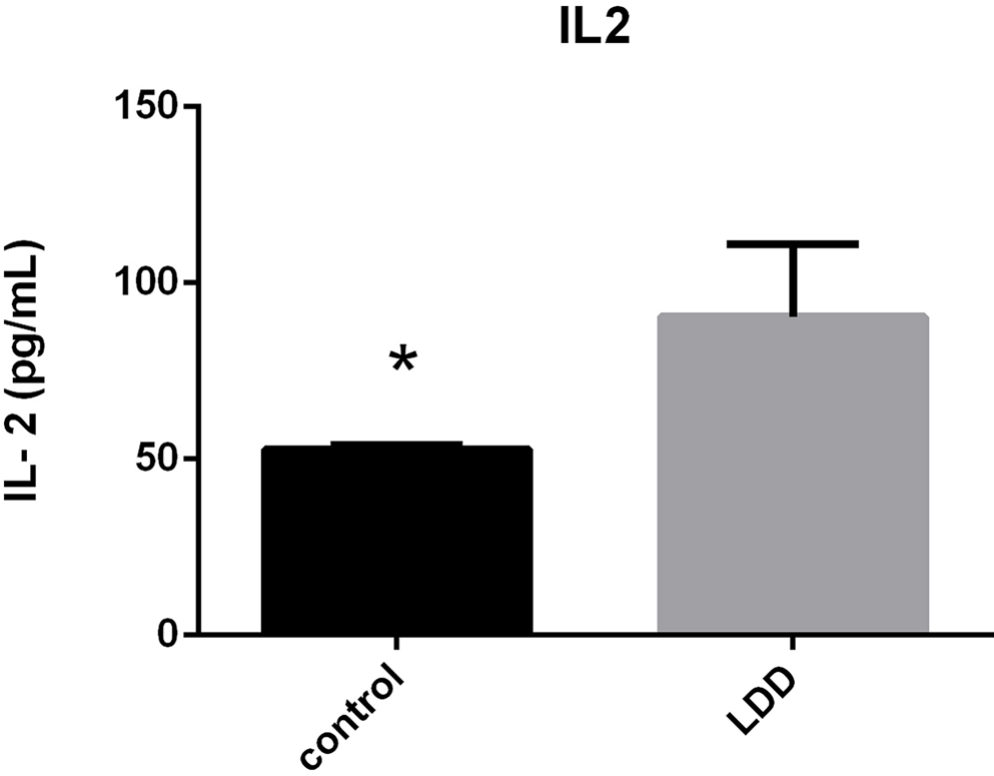
Serum IL-2 levels in LDDD patients and healthy controls are compared. The LDDD group had significantly higher levels of IL-2 (*p* < 0.001), which may indicate a systemic inflammatory profile

 Serum IL-2 concentrations were significantly higher in LDDD patients (101.7 ± 29.6 pg/mL) compared with controls (53.1 ± 9.7 pg/mL, *p* < 0.001) (Fig. 1). These elevated levels indicate a heightened inflammatory state in patients with disc degeneration.

### IL-2 variation by disease severity

**Fig. 2:**
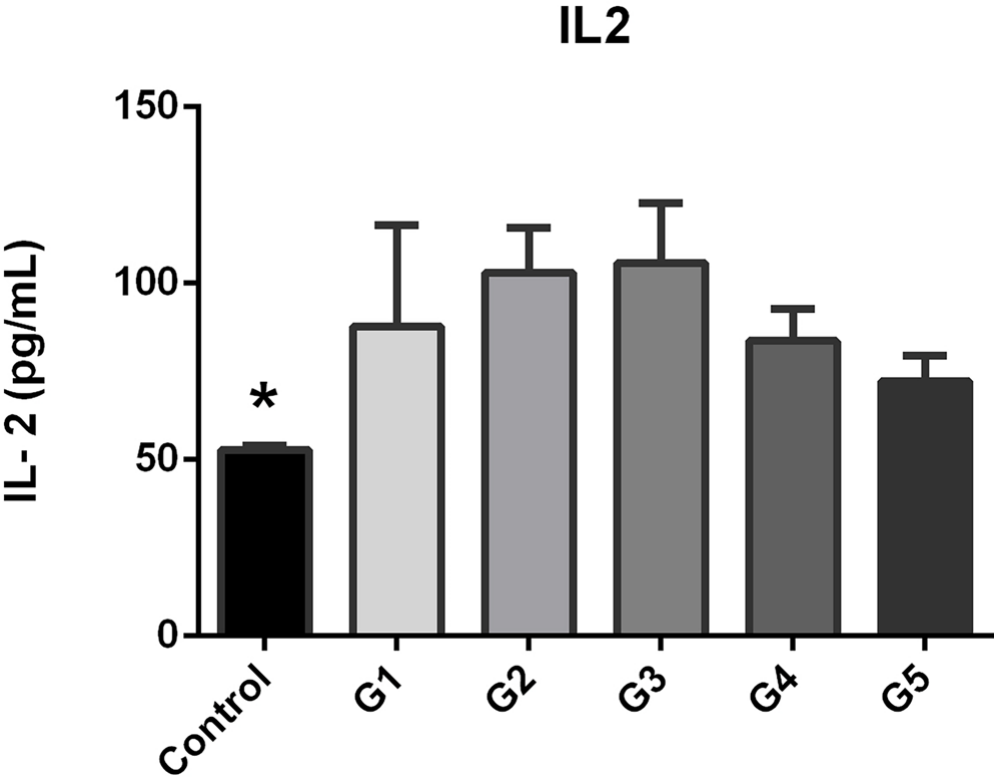
The serum IL-2 levels for each Oswestry Disability Index (ODI) subgroup. The highest inflammatory activity occurred during moderate disease severity, as indicated by IL-2 concentrations that peaked in Grades 2 and 3 and declined in advanced stages

IL-2 levels peaked in Grades 2 and 3 (108.3 ± 26.5 pg/mL and 106.4 ± 23.9 pg/mL, respectively) and declined in Grades 4 and 5. Grade 1 showed moderate elevation (84.1 ± 34.7 pg/mL) relative to controls (Fig. 2). These results suggest that inflammatory activity intensifies in the early and moderate stages of LDDD before subsiding in advanced disease.

### miR-186-5p expression in LDDD

**Fig. 3:**
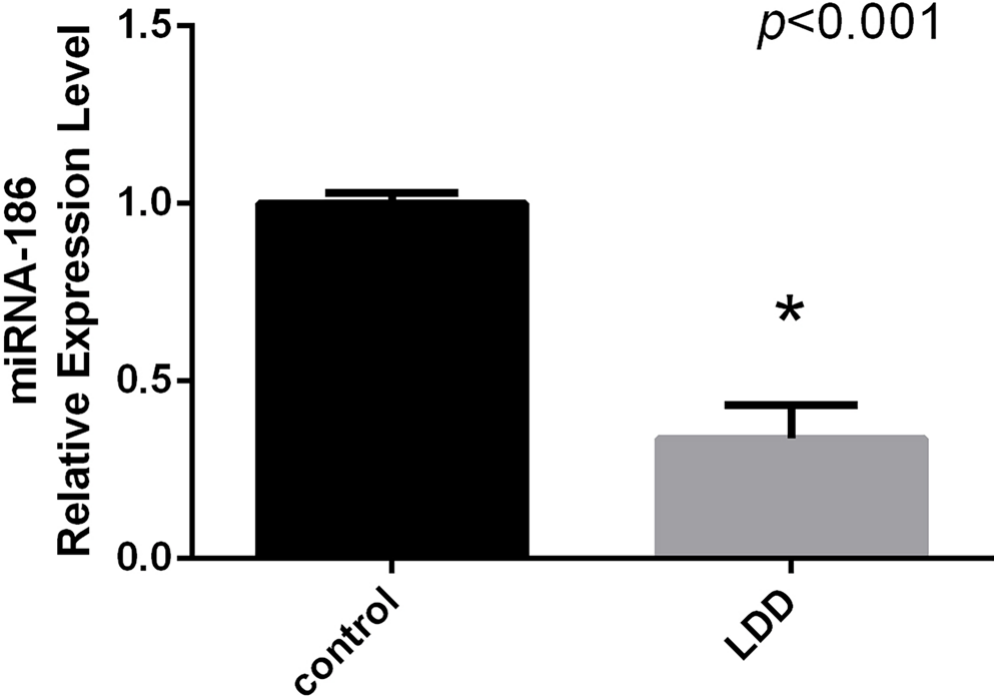
HSA-miR-186-5p expression in LDDD patients and healthy controls is compared. The patient group’s expression was significantly downregulated (*p* < 0.001), which is consistent with a loss of post-transcriptional anti-inflammatory control

 HSA-miR-186-5p expression was significantly lower in LDDD patients (0.31 ± 0.12) than in healthy controls (1.00 ± 0.09, *p* < 0.001) (Fig. 3). This finding indicates a clear downregulation of the anti-inflammatory miRNA in degenerative conditions.

### miR-186-5p expression across ODI grades

**Fig. 4:**
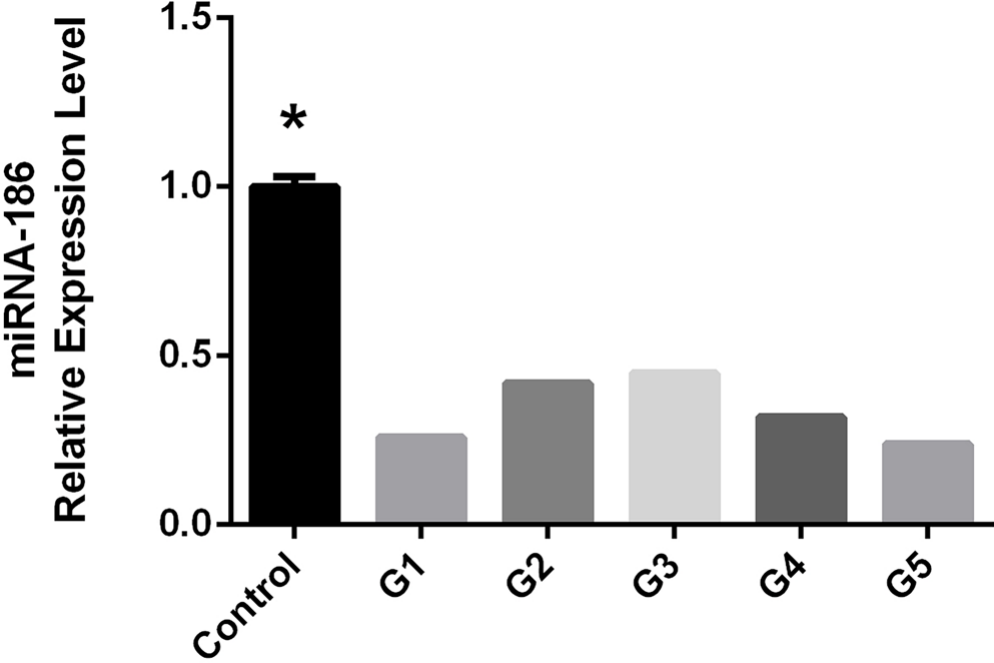
The relative expression levels of HSA-miR-186-5p in each ODI subgroup. With the highest expression in Grade 3 and the lowest in Grades 1 and 5, a biphasic pattern was seen, suggesting dynamic regulation as the disease progressed

 Subgroup analysis revealed that Grades 1 and 5 had the lowest miR-186-5p expression (0.27 ± 0.05 and 0.28 ± 0.04, respectively), while Grade 3 exhibited the highest level (0.49 ± 0.06) (Fig. 4). This biphasic pattern reflects dynamic miRNA regulation throughout disease progression.

### Correlation between IL-2 and miR-186-5p

**Fig. 5:**
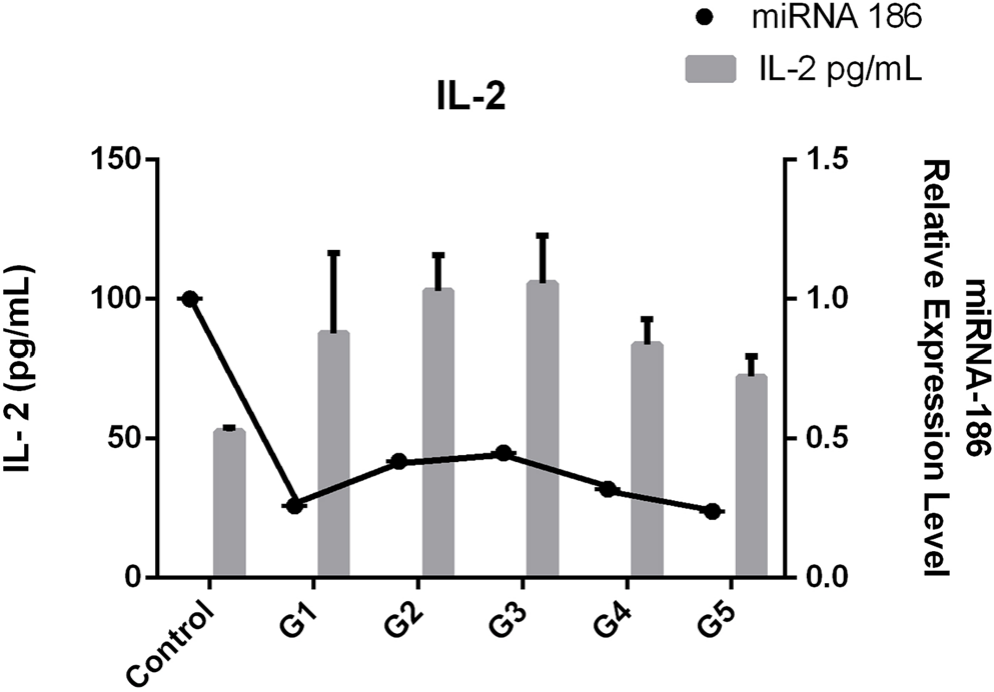
The inverse relationship between miR-186-5p expression and IL-2 levels across ODI subgroups. This lends credence to a possible regulatory interaction in LDDD pathophysiology

 A combined analysis of IL-2 and miR-186-5p across subgroups revealed an inverse relationship: as IL-2 increased, miR-186-5p decreased.This negative correlation supports the hypothesis that miR-186-5p acts as a regulator of IL-2-mediated inflammatory activity in LDDD (Fig. 5).

Overall, the findings indicate that reduced miR-186-5p expression and elevated IL-2 levels are associated with LDDD progression. These biomarkers may provide insight into the molecular mechanisms driving inflammation and degeneration in spinal pathology.

## Discussion

Chronic inflammation plays a central role in lumbar degenerative disc disease (LDDD), contributing to extracellular matrix breakdown, pain sensitization, and neural alterations. IL-2, typically linked to T-cell activation, has also been associated with degenerative disorders due to its ability to sustain immune activation in non-lymphoid tissues [4]. In this study, serum IL-2 levels were significantly elevated in LDDD patients, with the highest levels in moderate stages (ODI Grades 2–3). These findings suggest that inflammatory activity peaks during mid-stage degeneration.

MicroRNAs (miRNAs) are post-transcriptional regulators that fine-tune gene expression by binding to target mRNAs and modifying their stability or translation [5]. Dysregulation of miRNAs has been reported in several inflammatory and degenerative disorders, including spinal diseases [1]. In our cohort, miR-186-5p expression was markedly decreased in all LDDD subgroups, exhibiting a biphasic pattern—lowest in early (Grade 1) and late (Grade 5) disease, with a moderate rise in mid-stages. This dynamic expression may represent early downregulation leading to cytokine-driven inflammation, followed by further decline in advanced disease as fibrosis and neurodegeneration dominate.

Similar stage-dependent miRNA changes have been reported in other degenerative conditions, such as osteoarthritis, where miR-186-5p downregulation increased IL-1β-induced chondrocyte injury through MAPK1 targeting [6]. In rheumatoid arthritis, miRNA modulation influenced TNF-α signaling [2]. These parallels suggest that miR-186-5p contributes to inflammatory regulation across multiple tissues.

Beyond spinal pathology, miR-186-5p plays diverse roles depending on the cellular context. In cardiac models, its inhibition worsened high glucose-induced damage in AC16 cardiomyocytes, demonstrating a protective function under metabolic stress [7]. Conversely, in neuronal ischemia–reperfusion injury, its overexpression promoted apoptosis by targeting IGF-1, highlighting context-dependent effects [8]. Such pleiotropic behavior indicates that miR-186-5p’s impact depends on the surrounding microenvironment. Additionally, evidence from regenerative orthopedics, such as extracorporeal shock wave therapy for fracture nonunion, shows that modulating molecular pathways can significantly influence tissue recovery [9]. These observations suggest that miR-186-5p modulation may serve not only to reduce inflammation but also to enhance disc tissue repair.

Our findings align with previous research on miRNA–cytokine networks in spinal disorders, including miR-17 regulation of TNF-α and IL-6 in LDDD [3] and TNF-α–mediated progression in chordomas [10]. The observed inverse correlation between miR-186-5p and IL-2 strengthens the hypothesis that miR-186-5p acts as a negative regulator of IL-2–driven inflammation. The partial recovery of miR-186-5p expression during mid-stage disease, coinciding with peak IL-2 levels, may represent a potential therapeutic window in which restoring miR-186-5p could mitigate inflammation and slow disc degeneration. This interpretation is consistent with evidence that modulating specific miRNAs can alter cytokine expression and disease progression in inflammatory models [2, 11].

## Limitations

 This study is cross-sectional and serum-based; therefore, local disc microenvironmental processes were not directly examined. Treatment history (e.g., NSAIDs, steroids) was not stratified and may have influenced cytokine or miRNA profiles. Moreover, no direct mechanistic assays or disc tissue analyses were performed, so functional validation of the miR-186-5p/IL-2 axis remains necessary. Future studies should include direct examination of HSA-2 expression in disc tissue to confirm serum–tissue correlation.

In conclusion, we identified a significant inverse association between IL-2 elevation and miR-186-5p reduction in LDDD, accompanied by a stage-dependent biphasic miRNA pattern. These results suggest that miR-186-5p acts as an anti-inflammatory regulator through IL-2 suppression, particularly during early and moderate disease stages. Longitudinal and tissue-based studies are warranted to validate these findings and explore the therapeutic potential of targeting the miR-186-5p/IL-2 axis in degenerative disc disease.

## Conclusion

This study offers new proof that patients with lumbar degenerative disc disease have significantly lower levels of HSA-miR-186-5p and higher levels of IL-2. The idea of a regulatory axis where miR-186-5p may inhibit IL-2-driven inflammatory signaling is supported by the observed inverse correlation between these two biomarkers. The immunological mechanisms behind disc degeneration are better understood thanks to these findings, which also show that miR-186-5p may be a target for RNA-based treatments in the future and a non-invasive biomarker. To investigate the therapeutic implications of modifying miR-186-5p activity in the treatment of LDDD, more functional and longitudinal research is necessary.

## Data Availability

The data generated during this study are included in the article. Additional data are available from the corresponding author on reasonable request.
